# Dynamic changes of urinary proteins in a focal segmental glomerulosclerosis rat model

**DOI:** 10.1186/1477-5956-12-42

**Published:** 2014-07-21

**Authors:** Mindi Zhao, Menglin Li, Xundou Li, Chen Shao, Jianrui Yin, Youhe Gao

**Affiliations:** 1 Department of Pathophysiology, National Key Laboratory of Medical Molecular Biology, Institute of Basic Medical Sciences, Chinese Academy of Medical Sciences/Peking Union Medical College, Beijing 100005, China

**Keywords:** Urine proteome, Animal model, Biomarker, Focal segmental glomerulosclerosis

## Abstract

**Background:**

In contrast to blood, which has mechanisms to maintain a homeostatic internal environment, urine is more likely to reflect changes in the body. As urine accumulates all types of changes, identifying the precise cause of changes in the urine proteome is challenging and crucial in biomarker discovery. To reduce the effects of both genetic and environmental factors on the urinary proteome, this study used a rat model of adriamycin-induced nephropathy resembling human focal segmental glomerulosclerosis (FSGS) development.

**Results:**

Urine samples were collected at before adriamycin administration and day3, 7, 11, 15 and 23 after. Urinary proteins were profiled by liquid chromatography coupled with tandem mass spectrometry (LC-MS/MS). Of 23 changed proteins with disease development, 20 have human orthologs, and 13 proteins were identified as stable in normal human urine, meaning that changes in these proteins are more likely to reflect disease. Fifteen of the identified proteins have not been established to function in FSGS development. Seven proteins were selected for verification in ten more rats as markers closely associated with disease severity by western blot.

**Conclusion:**

We identified proteins changed in different stages of FSGS in rat models, which may aid in biomarker development and the understanding of FSGS pathogenesis.

## Background

Biomarkers represent measurable changes associated with a physiological or pathophysiological process. In contrast to the blood, which has mechanisms to maintain a homeostatic internal environment, urine is more likely to reflect changes in the body and can be collected noninvasively [1,2]. Due to the site of the formation and regulation of urine, the urine proteome has been widely investigated in studies of kidney, bladder and prostate diseases [3]. As urine accumulates all types of changes, identifying the precise cause of changes in the urine proteome is challenging and crucial in biomarker discovery. Using animal models is the most efficient way to find the cause and effect relationship, because of the following reasons. (1) Using animal models can reduce the effects of genetic and environmental factors on the urine proteome as much as possible. (2) Exact starting of the disease is available, which is helpful in the identification of biomarkers for each stages including early detection. (3) It can avoid the effects of medications on the urine proteome because therapeutic measures for patients are inevitable.

With a significantly increasing frequency in the past 20 years, focal segmental glomerulosclerosis (FSGS) is currently a common cause of nephrotic syndrome and responsible for 15% end-stage renal disease [4]. Some FSGS patients require kidney transplantation, and approximately one-fourth develop recurrent disease in the allograft [5]. The pathogenesis of FSGS remains unclear, and podocyte effacement leading to the injury of the glomerular filtration barrier is considered a main characteristic. Proteinuria is a leading clinical manifestation of this disease [6]. Only renal biopsy can distinguish different types of nephrotic syndrome. However, FSGS is “focal” and “segmental”, so the diagnosis of FSGS is often more problematic and complex [7]. Biomarkers in the urine are needed for its early diagnosis and prognosis.

Adriamycin (ADR)-induced nephropathy is the most prototypical and commonly used experimental model of human primary FSGS [8]. Renal abnormalities induced by ADR resembled FSGS in humans. The histological changes of ADR-induced nephropathy are characterized by podocyte injury followed by glomerular sclerosis, tubulointerstitial inflammation and fibrosis [9]. Analyzing the urinary proteome at different stages and identifying candidate biomarkers related to disease development will lead to a better understanding of FSGS pathogenesis.

**Figure 1:**
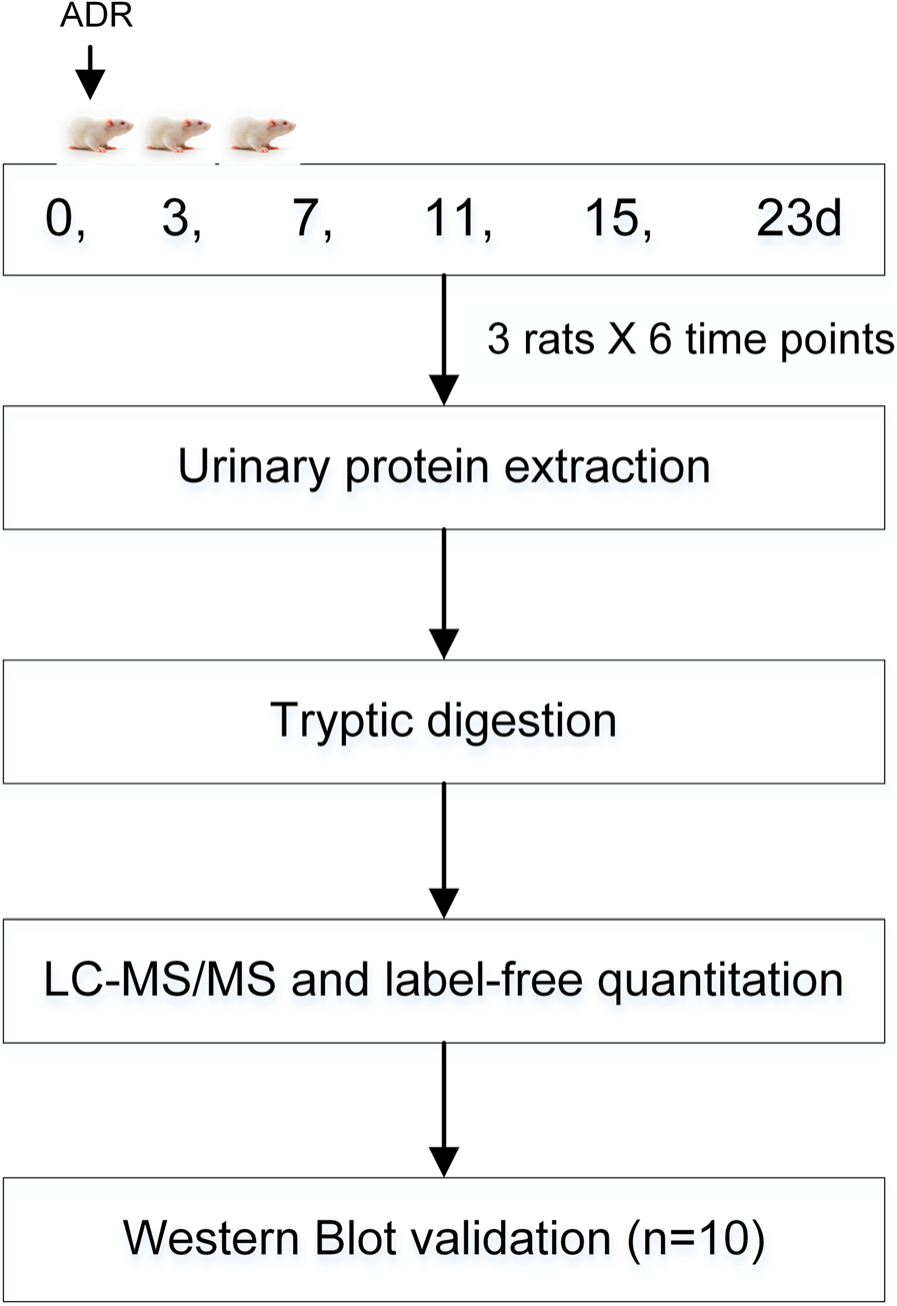
**Workflow of protein identification in the FSGS rat model.** Urine was collected at days 0, 3, 7, 11, 15 and 23 after ADR administration, and urinary proteins were enriched by ConA agarose followed by liquid chromatography coupled with tandem mass spectrometry (LC-MS/MS) identification. Several proteins were verified in ten more rats by western blot analysis.

In the late phase of FSGS, massive irreversible proteinuria commonly occurs after podocyte injury, which increases the difficulty of identifying some low-abundance proteins by MS. Concanavalin A (ConA) enrichment is frequently used to enrich for N-linked glycoproteins in both plasma and urine proteomic studies [10,11]. As many existing biomarkers are glycoproteins, in this study, ConA-enriched urinary protein was evaluated to monitor the dynamic changes during FSGS development (Figure 1).

## Results and discussion

### The urinary protein to creatinine ratios increased

**Figure 2:**
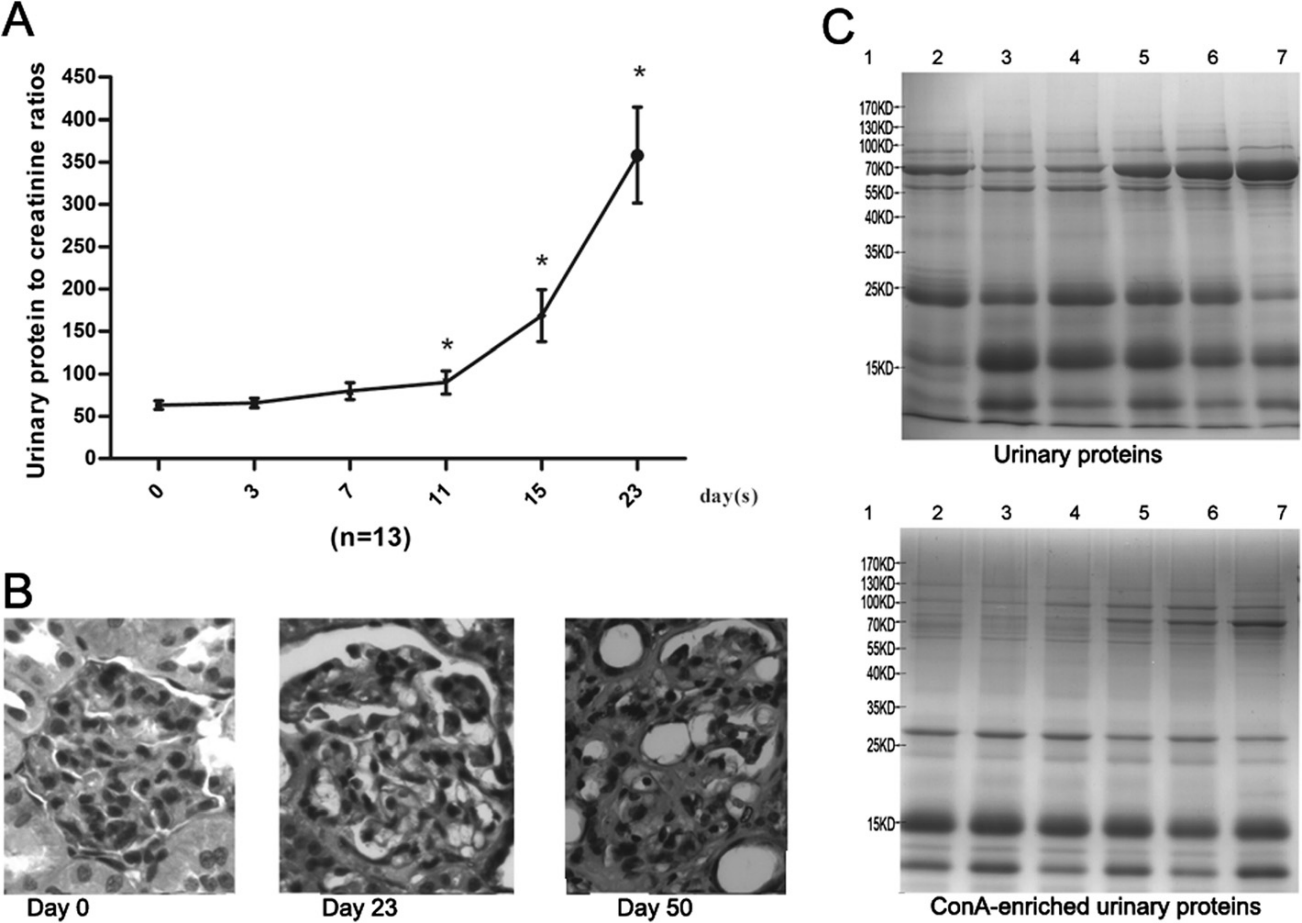
**Dynamic changes in ADR-treated rats. A)** Urinary protein to creatinine ratios in six phases of ADR treated rats. The data are expressed as the mean ± standard deviation (n = 13, *p < 0.05 for Experiment vs. Control). The paired t test was used to assess the significance of the differences between groups. **B)** Histological changes in ADR-treated rats (HE staining, 400×). Compared with the normal condition, on day 23, glomerular lesions with mesangial matrix expansion could be detected; on day 50, glomerular sclerosis and severe interstitial expansion were found in many glomeruli. **C)** Urinary protein and ConA-enriched urinary protein pattern of one FSGS model rat. Lane 1: Marker. Lanes 2–7, urinary proteins and ConA-enriched urinary proteins at days 0, 3, 7, 11, 15 and 23 respectively.

To monitor renal function, the urinary protein to creatinine ratios were measured to determine the proteinuria levels. As shown in Figure 2A, the ratios increased. From day 3 to day 7, the increase was moderate. Marked increases occurred 11 days after ADR induction. To confirm the successful induction of FSGS, the histological characteristics of the rat kidneys on days 23 and 50 were examined. On day 23, the glomerular endothelium and podocyte were partly effaced, foam cells were generated, and fewer glomerular lesions with mesangial matrix expansion were present. On day 50, severe segmental and global sclerosis, mesangial matrix expansion and interstitial fibrosis were present (Figure 2B). All the features indicated that the disease was in the late stage.The urinary proteins at days 0, 3, 7, 11, 15 and 23 were analyzed by SDS-PAGE. Compared with day 0, the expression of the 70-kD protein was gradually increased, and this protein became the most intense band at day 23. The ConA-enriched samples at each stage were also shown in Figure 2C.

### Dynamic urinary proteome changes were identified by LC-MS/MS

**Figure 3:**
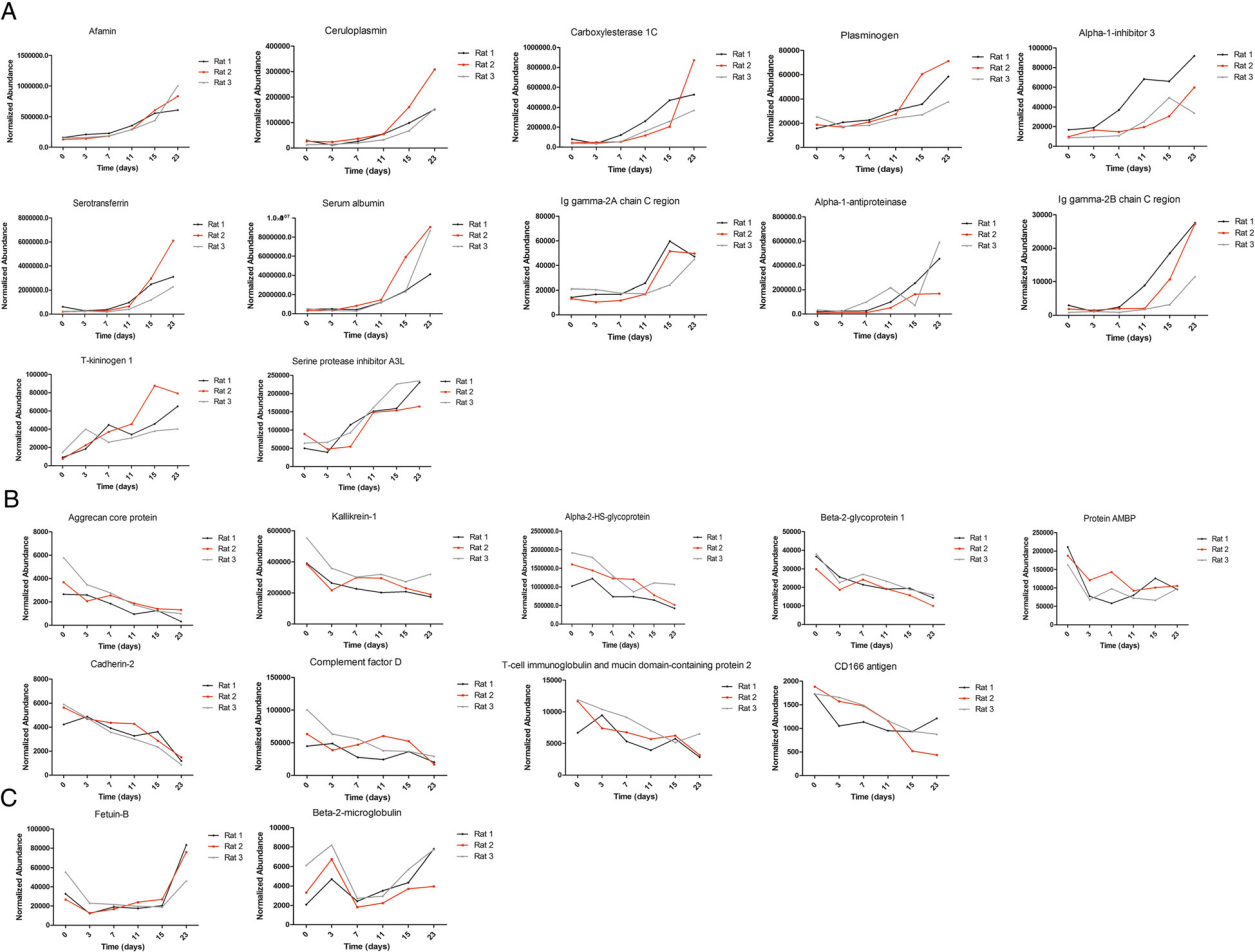
**Expression of candidate urine biomarkers of ADR-induced nephropathy during six stages. A)** Twelve proteins shared an overall increasing trend in relative abundance. **B)** Nine proteins shared an overall decreasing trend. **C)** Two proteins changed at the early stage. The x axis represents different stages; the y axis represents the normalized abundance identified by the LC-MS Progenesis software.

At the discovery stage, eighteen samples of three rats (six time points each) were individually analyzed by UPLC coupled with Triple-TOF 5600 MS. LC-MS Progenesis software was used to calculate the normalized abundance of each protein by measuring the peak area intensity. By label-free quantitative and statistical analyses, 23 proteins met the following criteria: (1) compared with day 0, fold change > 2 in each rat and (2) p value < 0.05 (data were analyzed by repeated measure ANOVA). Among the 23 changed proteins, 20 proteins were annotated as glycoproteins in the UniProt database, 12 proteins shared an overall increasing trend in relative abundance, and 9 proteins shared an overall decreasing trend (Figure 3). The changed proteins are listed in Additional file 1 and the label-free quantitation data are shown in Additional file 2.Three trends were observed in these changed proteins during ADR-induced nephropathy progression. The first was a gradual increase, with examples including afamin and ceruloplasmin. Urinary afamin increased only 1.16-fold on the third day after ADR injection, remained elevated compared with normal urine samples, and peaked at 5.63-fold on day 23 after the ADR injection. The second was a gradual decrease, with examples including cadherin-2 and aggrecan core protein. Cadherin-2 decreased 1.1-fold after 3 days and continued to decrease in the following days. The third, which includes fetuin-B and beta-2-microglobulin, was early changes with distinct patterns. Fetuin-B decreased 2.39-fold on day 3 and remained unchanged during the following days until increasing 1.79-fold on day 23. Beta-2-microglobulin increased 1.7-fold on day 3, decreased 2.83-fold on day 7, and gradually increased 1.69-fold on day 23, compared with the normal samples. The time-dependent changes of 23 representative proteins from three rats are shown in Figure 3.

At the early stage, such as 3 and 7 days after the ADR injection, proteinuria was not obvious. However, several proteins, such as fetuin-B, AMBP and alpha-2-HS-glycoprotein, were decreased during this phase. These proteins may be good candidates for the early detection of FSGS.

### Western blot validation

**Figure 4:**
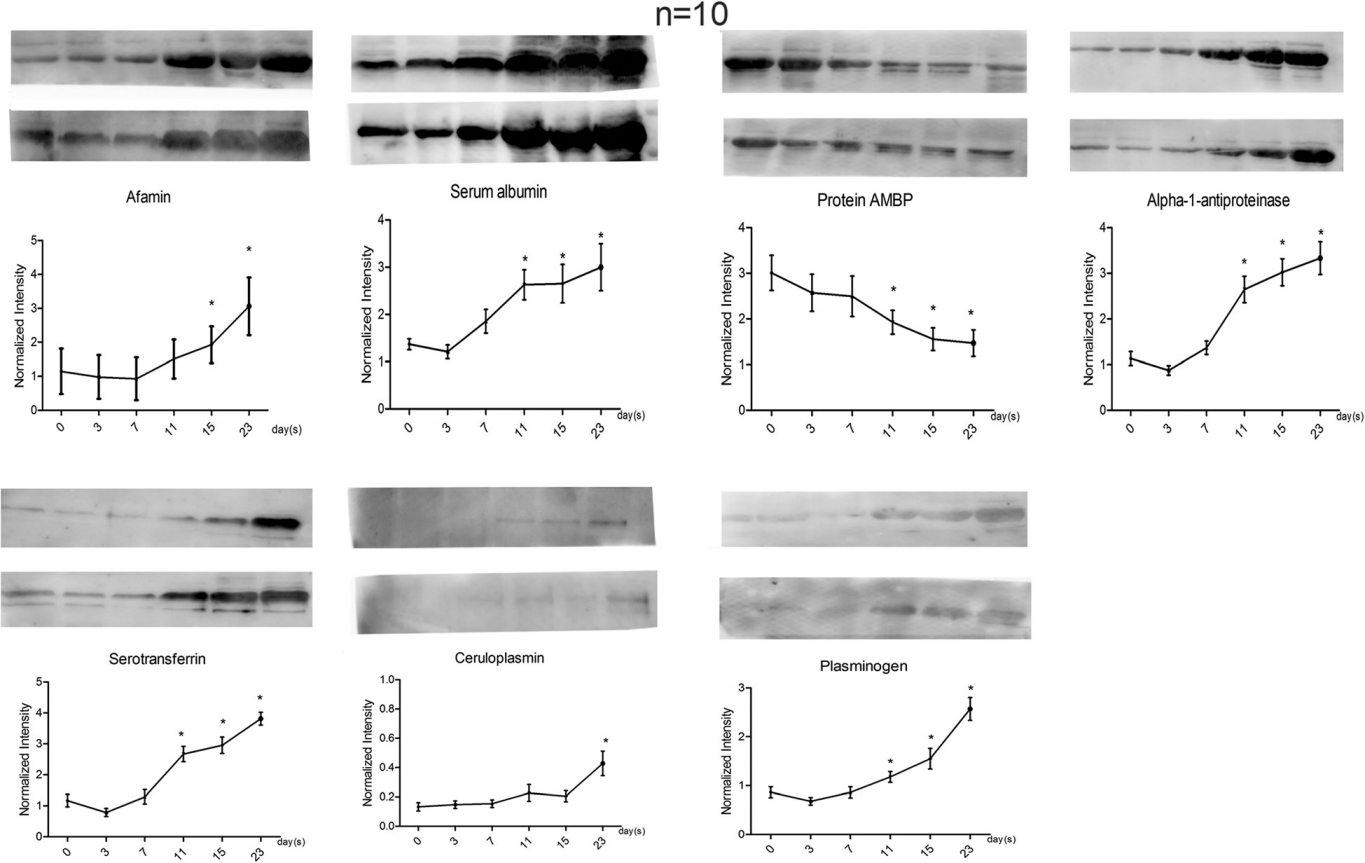
**Semi-quantitative western blot analysis of seven proteins.** Semi-quantitation of albumin, serotransferrin, alpha-1-antiproteinase, afamin, ceruloplasmin, plasminogen and AMBP determined by western blot analysis from ten additional rats at six time points each. The two western blots for each protein shown are representative of the group of ten.

The urine samples from ten more rat models at six time points each were collected for validation. Of 23 changed proteins with disease development, nine altered proteins (albumin, serotransferrin, alpha-1-antiproteinase, afamin, ceruloplasmin, plasminogen, kininogen, alpha-2-HS-glycoprotein and alpha 1 microglobulin [AMBP]) identified in our proteomic study with human orthologs at relatively high abundance that also had commercially available antibodies were selected to be analyzed by semi-quantitative western blot analysis (Figure 4). Kininogen-1 and alpha-2-HS-glycoprotein were not detected by western blot. The changes in the other seven proteins were consistent with the LC-MS/MS results.

### Human orthologs of changed proteins

Changed proteins demonstrated in animal models are more valuable if they can be confirmed in humans. The changed proteins were converted to their corresponding human orthologs using Ensembl Gene ID(s) by Ensembl BioMart (http://asia.ensembl.org/biomart/martview) as described [12,13]. Of 23 identified changed proteins, 20 have human orthologs, including the seven validated by western blot analysis.

Urinary proteins are mainly composed of plasma proteins filtered by the glomerular barrier and proteins secreted from the kidney and urinary tract [14]. A previous study compared the kidney input (plasma) and output (urine) proteomes and divided urinary proteins into 3 categories, the plasma-only subproteome, the plasma-and-urine subproteome, and the urine-only subproteome [15]. To further analyze the functions of these changed proteins, they were compared with the human plasma proteome, human urine proteome and kidney origin proteome. The human proteome data were downloaded from the Healthy Human Individual’s Integrated Plasma Proteome [16], the human urine proteome data were acquired from previous studies [17-19], and the kidney origin proteome data were acquired from a kidney perfusion study [13]. The human orthologs of the changed proteins and their relationships with the human plasma, urine and kidney origin proteomes are shown in Table 1. Most changed proteins exist in the normal human plasma proteome (17/20) and urine proteome (18/20); however, the CD166 antigen was detected only in the plasma proteome, kallikrein-1 was detected only in the normal urine proteome, and 10 proteins were detected in the kidney origin proteome. In addition, the urinary proteome is largely affected by individual factors, but biomarkers should be applicable to most people, in other words, changes in the stable contents of the healthy human urinary proteome are more likely to be biomarkers [20]. A total of 560 proteins were considered stable in healthy human urine [21]. Thirteen out of twenty changed proteins in this study could be found among the stable proteins, indicating that these changed proteins were more useful for clinical diagnoses as they are not affected by individual differences and are not time-dependent (Table 1).

**Table 1:** Comparison of human orthologs of changed proteins with urine, plasma and kidney origin proteome

**Corresponding human protein ID**	**Protein name**	**Plasma proteome**	**Urine proteome**	**Kidney origin proteome**	**Stable protein**	**Candidate biomarkers of diseases**
P01009	Alpha-1-antiproteinase	Yes	Yes	Yes	Yes	FSGS [22]
P43652	Afamin	Yes	Yes	Yes	Yes	Diabetic nephropathy [23]
P02768	Serum albumin	Yes	Yes	Yes	Yes	Adriamycin nephropathy mice model [24]
P00450	Ceruloplasmin	Yes	Yes	Yes	Yes	Diabetic nephropathy [25]
P01042	Kininogen-1	Yes	Yes	Yes	Yes	Adriamycin nephropathy rat model [26]
P02787	Serotransferrin	Yes	Yes	Yes	Yes	Adriamycin nephropathy mice model [24]
P23141	Carboxylesterase 1	Yes	Yes	No	No	None
P01861	Ig gamma-4 chain C region	Yes	Yes	No	No	Kidney calculi [27]
P00747	Plasminogen	Yes	Yes	No	Yes	Acute appendicitis [28]
P02765	Alpha-2-HS-glycoprotein	Yes	Yes	Yes	Yes	Diabetic nephropathy [23]
P02760	Protein AMBP	Yes	Yes	Yes	Yes	FSGS [22]
Q96D42	T-cell immunoglobulin and mucin domain-containing protein 1	No	No	No	No	Renal cell carcinoma [29], acute renal failure [30]
P19022	Cadherin-2	Yes	Yes	No	Yes	Ureteropelvic junction obstruction [31]
Q6PID9	Aggrecan core protein	No	Yes	No	No	None
P00746	Complement factor D	Yes	Yes	No	No	Dents disease [32]
P02749	Beta-2-glycoprotein 1	Yes	Yes	No	Yes	Dents disease [32]
Q13740	CD166 antigen	Yes	No	No	No	None
P06870	Kallikrein-1	No	Yes	No	Yes	None
P61769	Beta-2-microglobulin	Yes	Yes	Yes	Yes	Renal Interstitial Inflammation [33]
Q9UGM5	Fetuin-B	Yes	Yes	Yes	No	None

Changed proteins identified by progressive ADR-induced nephropathy were compared with a manually curated human and animal urinary protein biomarker database [34]. Five high-abundance urinary proteins, including albumin, serotransferrin and kininogen-1, had been present in higher levels in previous FSGS studies [22,24,26], which is consistent with the results of this study. The other 15 proteins were not found to be related to FSGS. Some proteins were found to be candidate biomarkers for other glomerular diseases; for example, elevated afamin expression has been reported in some diabetic nephropathies. The similarities may due to a common pathway of different glomerular diseases at later stages [35]. Thus, these diseases can be differentiated using changed proteins, especially those significantly decreased proteins in the early stage, such as fetuin-B and AMBP. Some candidates also appeared in other renal diseases, whereas the opposite trends were observed in different diseases. For example, complement factor D and beta-2-glycoprotein 1 were higher in Fanconi syndrome [36] but lower in ADR-induced nephropathy.

As shown in Table 1, the same urinary proteins appeared to be modulated in several renal diseases. For example, albumin was increased in both diabetic nephropathy and IgA nephropathy. Thus, it may be difficult to provide an accurate diagnosis using a single biomarker; a panel of urinary proteins may be more specific and more sensitive. In this study, the 20 proteins that have human orthologs could be evaluated together to reflect the progression of FSGS. We hope this work will help to support the role of urine as a key source in biomarker discovery especially in kidney diseases and help to identify FSGS biomarkers for clinical utility.

As urine proteome is subjected to many factors like diet, drugs and lifestyles, increasing the number of clinical samples is laborious and almost futile with current sample analysis throughput. We think finding clues from well controlled animal experiment and validating them in clinical samples is a better way to develop biomarkers in urine [37]. The advantage of ConA enrichment in this study is not as significant as we expected, but it did not change the main idea of the paper. If these high abundant protein panels identified in this study are not sufficiently specific and sensitive to distinguish renal diseases clinically, the identification of less abundant proteins in the discovery phase will be necessary in future studies.

## Conclusions

In this study, we set to observe the urine proteome changes in rat models as the disease progress, which may present a possible direction to break through the current stalemate in urinary biomarker field. The results showed proteins changed in different stages of FSGS, which may aid in biomarker development and the understanding of FSGS pathogenesis.

## Methods

### Experimental animals

Rats were purchased from the Institute of Laboratory Animal Science, Chinese Academy of Medical Science & Peking Union Medical College. The experiment was approved by Institute of Basic Medical Sciences Animal Ethics Committee, Peking Union Medical College. All animals were maintained with a standard laboratory diet under controlled indoor temperature (22 ± 1°C) and humidity (65 – 70%) conditions. The study was performed strictly according to the guidelines developed by the Institutional Animal Care and Use Committee of Peking Union Medical College. All efforts were made to minimize suffering.

### Rat model of FSGS induced by adriamycin

Young Sprague–Dawley male rats (n = 13, initial weight 200 g) were given a single intravenous injection of ADR (5 mg/kg). Urine samples were collected at days 0 (before injection), 3, 7, 11, 15 and 23 after the rats were placed in metabolic cages 4 hours individually. During urine collection, to avoid contamination, all rats were given free access to water but no food. After sample collection, six rats were sacrificed on day 23, and the remainder were sacrificed on day 50. The 13 harvested kidneys were subjected to histopathological examination. The urinary proteins to creatinine ratio was evaluated to determine the proteinuria level [38]. The self-controlled experiment was conducted in two phases: for the discovery phase, differential protein identification was performed in three independent rats; for the validation phase, samples were obtained from the ten remaining rats, with each animal providing six urine samples.

### ConA enrichment of urinary proteins

Once collected, the urine was centrifuged at 2000 g for 15 min. After removing the cell debris, the supernatant was centrifuged at 12 000 g for 15 min at 4°C. Urinary proteins were extracted by acetone precipitation from the individual urine samples. Then, 25 mM NH_4_HCO_3_ was used to re-dissolve the pellets after centrifugation at 12 000 g. The protein concentration of each sample was measured by the Bradford method.

The enrichment of ConA-binding urinary proteins was conducted in the three rats that were used in the proteomic analysis, as described previously [39]. The individual samples from each time point were incubated with ConA agarose at 4°C overnight. To remove non-specific ConA-binding proteins, the sample-loaded beads were washed with Buffer A (150 mM NaCl, 1 mM CaCl_2_, 1 mM MgCl_2_, 20 mM Tris, pH 7.4) three times. The target proteins were eluted with 500 mM methyl α-D-glucopyranoside in buffer A.

To examine the differences between urinary proteins and ConA-enriched proteins, SDS-PAGE was used to separate the proteins. 20 μg of urinary proteins at different stages of FSGS and 5 μg of their corresponding ConA-enriched proteins were loaded onto 20*12-cm 12% SDS-PAGE. The gels were stained with Coomassie Brilliant Blue.

### Tryptic digestion

The proteins were digested with trypsin (Trypsin Gold, Mass Spec Grade, Promega, Fitchburg, WI) using filter-aided sample preparation methods [40]. After 100 μg of ConA-enriched proteins were loaded on the 10-kD filter devices (Pall, Port Washington, New York, USA), 200 μl of UA (8 M urea in 0.1 M Tris–HCl, pH 8.5) was added twice to wash the proteins, and the tube was centrifuged at 14 000 g for 20 min at 18°C. The proteins were denatured by incubating with 5 mM dithiothreitol (DTT) at 50°C for 1 h and then alkylated in the dark for 45 min by the addition of 50 mM iodoacetamide (IAA). The resulting proteins were diluted in 300 μl of UA and centrifuged twice. The concentrate was redissolved in 50 mM NH_4_HCO_3._ Then, trypsin was added (enzyme to protein ratio of 1:50) and incubated at 37°C overnight. The digested peptides were collected by centrifugation and sequential incubation with PNGase F (New England Biolabs, USA) overnight. The filtrates were desalted using Oasis HLB cartridges (Waters, Milford, MA) and then dried by vacuum evaporation (Thermo Fisher Scientific, Bremen, Germany).

### LC-MS/MS analysis

The digested peptides were dissolved in 0.1% formic acid and transferred to a reversed-phase microcapillary column using a Waters UPLC system. The peptides were separated on a 10-cm fused silica column. Elution was performed over a gradient of 5%–28% buffer B (0.1% formic acid, 99.9% ACN; flow rate, 0.3 μL/min). The MS data were acquired using the AB SCIEX (Framingham, MA, US) Triple-TOF 5600 mass spectrometer system. Three biological replicates were performed in this study, with each having six samples.

### Protein identification and label-free quantitation

The MS/MS data were processed using Mascot software (version 2.4.1, Matrix Science, London, UK) and searched against the Swissprot_2013_05 database (taxonomy: Rattus; containing 9,354 sequences). For peptide identification, the fragment ion mass tolerance was set to 0.05 Da, and the parent ion tolerance was set to 0.05 Da. The search allowed two missed cleavage sites in the trypsin digestion. The carbamidomethylation of cysteines was considered a fixed modification, and the oxidation of methionine and deamidation of asparagine were considered variable modifications. Peptide and protein identifications were validated by Scaffold (version 4.0.1, Proteome Software Inc., Portland, OR). Peptide identifications were accepted if they could be established to achieve an FDR less than 1%. Protein identifications were accepted if they could be established at greater than 95.0% probability. The label-free quantification was conducted as previously described [41] using Progenesis LC-MS/MS software (version 4.1, Nonlinear, Newcastle upon Tyne, UK). Briefly, the acquired data of the MS scans were transformed and stored in peak lists using a proprietary algorithm. Features with only one charge or more than five charges were excluded from the analyses. Protein abundance is calculated from the sum of all unique peptide ion abundances for a specific protein on each run. Normalisation of abundance is required to allow comparisons across different sample runs by this software. For further quantitation, all peptides (with Mascot score > 20 and p < 0.01) of an identified protein were included. Proteins identified by more than one peptide were retained.

### Western blot verification

A total of 20 μg of urinary proteins from individual samples (n = 10, six samples per rat) were loaded on 10% SDS-PAGE and transferred to PVDF membranes. After blocking in 5% (w/v) skim milk for 1 h at room temperature, the membranes were incubated with the primary antibodies overnight at 4°C. The antibodies used for the analysis included albumin (anti-albumin, 1:1000, ab106582, Abcam, Cambridge, U.K.), serotransferrin (anti-serotransferrin, 1:10000, ab82411, Abcam, Cambridge, U.K.), alpha-1-antiproteinase (anti-alpha-1-antiproteinase, 1:10000, ab106582, Abcam, Cambridge, U.K.), afamin (anti-afamin, 1:300, sc-74311, Santa Cruz Biotechnology, Santa Cruz, CA), ceruloplasmin (anti-ceruloplasmin, 1:1000, ab131220, Abcam, Cambridge, U.K.), plasminogen (anti-plasminogen, 1:10000, ab6189, Abcam, Cambridge, U.K.), T-kininogen 1 (anti- kininogen 1, 1:300, sc-103886, Santa Cruz Biotechnology, Santa Cruz, CA), alpha-2-HS-glycoprotein (alpha-2-HS-glycoprotein antibody, 1:300, sc-20872, Santa Cruz Biotechnology, Santa Cruz, CA) and AMBP (anti-alpha 1 microglobulin, 1:1000, ab110707, Abcam, Cambridge, U.K.). The membranes were then washed with TBST three times and incubated with horseradish peroxidase-conjugated secondary antibody. Immunoreactive proteins were visualized using enhanced chemiluminescence (ECL) reagents. ECL results were scanned and analyzed using an ImageQuant 400TM Imager (GE Healthcare Life Sciences, Piscataway, New Jersey, USA) and the intensity of each protein band was quantified using Image J analysis software (National Institutes of Health, Bethesda, Maryland, USA).

## Competing interests

The authors declare that there are no competing interests.

## Authors’ contributions

MZ and YG designed the experiment; MZ performed the experiments, analyzed data and wrote the manuscript; ML and XL performed the LC–MS/MS analysis; CS and JY analyzed the data. All authors read and approved the final manuscript.

## Supplementary Material

Additional file 1 The changed proteins identified in all samples. Click here for file

Additional file 2 The label-free quantitation data of proteins identified in each sample. Click here for file
